# The screening assessment of pro-inflammatory, anti-inflammatory, Th1, Th2 and Th17 cytokines in saliva of patients with ischemic stroke

**DOI:** 10.7150/ijms.110452

**Published:** 2025-05-28

**Authors:** Dominika Forszt, Karolina Gerreth, Jakub Kopczyński, Anna Zalewska, Katarzyna Hojan, Renata Marchewka, Marzena Bielas, Mateusz Maciejczyk

**Affiliations:** 1 Department of Risk Group Dentistry, Pediatric Dentistry, Poznan University of Medical Sciences, Poznan, Poland; 2 Department of Risk Group Dentistry, Chair of Pediatric Dentistry, Poznan University of Medical Sciences, Poznan, Poland; 3 Students Scientific Club “Biochemistry of Civilization Diseases” at the Department of Hygiene, Epidemiology and Ergonomics, Medical University of Bialystok, Poland;; 4 Experimental Dentistry Laboratory, Department of Conservative Dentistry, Medical University of Bialystok, Bialystok, Poland; 5 Department of Occupational Therapy, Poznan University of Medical Sciences, Poznan, Poland; 6 Department of Rehabilitation, Greater Poland Cancer Centre, Poznan, Poland; 7 Neurorehabilitation Ward, Greater Poland Provincial Hospital, 60-480 Poznan, Poland;; 8 Department of Family Medicine, Poznan University of Medical Sciences, Poznan, Poland;; 9 Department of Hygiene, Epidemiology and Ergonomics, Medical University of Bialystok, Bialystok, Poland

**Keywords:** biomarkers, saliva, stroke, cytokines

## Abstract

**Introduction:** Ischemic stroke leads to hypoxia of brain structures, causes inflammation and tissue necrosis. Since the blood-brain barrier is damaged, inflammatory mediators can enter the bloodstream and saliva. This case-control study examines pro-inflammatory, anti-inflammatory, Th1, and Th2 cytokines in the unstimulated saliva of stroke patients and explores the association between these biomarkers and clinical status.

**Patients and methods:** The study group included 22 patients with ischemic stroke, while the control group included 22 healthy individuals that were matched for age, sex, dental, periodontal and oral hygiene status to the study group. Unstimulated saliva was collected from each patient. In each sample, the inflammatory profile was determined using the multiplex ELISA method. Due to the lack of normal distribution, the Mann-Whitney U test was used for comparisons. The research was approved by the Bioethics Committee of the Poznan University of Medical Sciences (59/19, 890/19, 504/21).

**Results:** In the unstimulated saliva of stroke patients, significantly higher levels of pro-inflammatory cytokines (IL-1β (p=0.0003), TNF-α (p≤0.0001), TNF-β; (p≤0.0001)), anti-inflammatory cytokines (IL-1ra (p≤0.0001), TRAIL (p=0.0206)), Th1 cytokines (IFN-**γ** (p≤0.0001), IL-2Rα (p=0.0021) and IL-12 (p40) (p≤0.0001)) and Th2 cytokines (IL-6 (p=0.0023)) were found compared to healthy participants. Interestingly, Addenbrooke's Cognitive Examination Revised (ACE-R) correlated negatively with TNF-α (p=0.01, r=-0.53), TNF-β (p=0.02, r=-0.60) and IFN-γ (p=0.02, r=-0.50). The Functional Independence Measure (FIM) exhibited a positive association with IL-6 (p=0.003, r=0.62) and BI (p=0.001, r=0.66). The positive correlation was found between Sitting Balance Scale (SBS) and IL-6 (p=0.02, r=0.52).

**Conclusions:** Stroke patients exhibit altered salivary composition characterized by increased secretion of inflammatory mediators. The obtained results do not indicate the dominance of any of the branches of the immune response. The concentration of salivary TNF-α, TNF-β, IFN-**γ** and IL-12 significantly distinguish patients with ischemic stroke from healthy individuals. Although validation of the results in a larger patient population is necessary, salivary cytokines show potential as diagnostic biomarkers.

## Introduction

Stroke is a civilization disease that poses a very serious social problem. Epidemiological studies indicate that stroke is the second leading cause of death 1 and the third cause of disability in developed and developing countries 2. Therefore, new therapeutic and diagnostic methods are being explored to improve outcomes for stroke patients.

Approximately 87% of strokes result from blocked blood supply, leading to ischemic stroke 3. The damage occurs in the part of the brain that is supplied by the affected vessel. Reduced blood flow deprives neurons of oxygen and nutrients, ultimately leading to their necrosis 4. Ischemia triggers a cascade of processes that occur at the cellular level, including electrolyte imbalances, acidosis, ATP depletion, excessive glutamate release and free radical overproduction. As a consequence, apoptosis, excitotoxicity, edema and inflammation of the brain occur 5. The primary goal of inflammation is to clear dead cells and facilitate synapse regeneration through cytokine secretion 6. However, long-term inflammation can cause brain damage, which further worsens the effects of stroke 7. Inflammation is induced by the release of high-mobility group box 1 (HMGB1) proteins from necrotic cells, which bind to receptors for advanced glycation end products (RAGE) 8, 9. The HMGB1-RAGE pathway plays an important role in local activation of the inflammatory response in microglia 9, 10. The inflammatory response is amplified by neutrophils, macrophages, and lymphocytes arriving from the systemic circulation, as well as dendritic cells accumulating at the periphery of the ischemic region 6. The activity of these cells triggers inflammation and endothelial cell apoptosis in the brain, increasing blood-brain barrier (BBB) permeability 11,12. Damage to the BBB allows inflammatory mediators to move from the brain into the bloodstream 13.

Saliva is a plasma filtrate, and due to the salivary glands' rich vascularization, it allows numerous blood biomarkers to enter the saliva 14. The use of this diagnostic material offers numerous benefits, such as easy and non-invasive sample collection, as well as convenient transport and storage 15. In our previous studies, we demonstrated the diagnostic potential of salivary tumor necrosis factor α (TNF-α), interleukin 6 (IL-6), interleukin 10 (IL-10) and xanthine oxidase (XO) in the differential diagnosis of stroke patients. Salivary TNF-α correlated with cognitive decline and the severity of functional impairments 16, 17. However, other inflammatory biomarkers have not been assessed in the saliva of stroke patients. It is well known that cytokines Th1, Th2, and Th17 play an important role in stroke. This study aims to characterize the salivary cytokine profile in stroke patients and explore the relationship between these biomarkers and clinical parameters.

## Patients and Methods

### Ethics Committee

The research was approved by the Bioethics Committee of the Poznan University of Medical Sciences (59/19, 890/19, 504/21) confirming the principles of the Declaration of Helsinki on human experimentations. Each patient signed an informed consent form after receiving detailed information about the purpose and scope of the study.

### Study Group

The patients qualified for this study were staying at a Neurorehabilitation Ward at the Greater Poland Provincial Hospital (Kiekrz, Poland), which provides comprehensive rehabilitation assistance for individuals with neurological problems. Patients were enrolled into the study between August 2021 and May 2022.

The inclusion criteria required radiological confirmation of ischemic stroke based on the World Health Organization (WHO) standards 18-20, the first stroke in a patient's life, age over 18 years, ability to give informed consent for the study and saliva sample collection, and the ability to provide a saliva sample. The exclusion criteria included a history of second or subsequent stroke, autoimmune (e.g., diabetes, Hashimoto's disease, psoriasis, rheumatoid arthritis, systemic sclerosis) or psychiatric diseases (e.g., Alzheimer's disease, Parkinson's disease), prior acute coronary events, thromboembolic events (such as limb thrombosis or pulmonary embolism), smoking, inability to give informed consent, or to provide a saliva sample. A general medical history, including information on chronic diseases, smoking, education and lifestyle, was collected from each patient.

**Figure 1:**
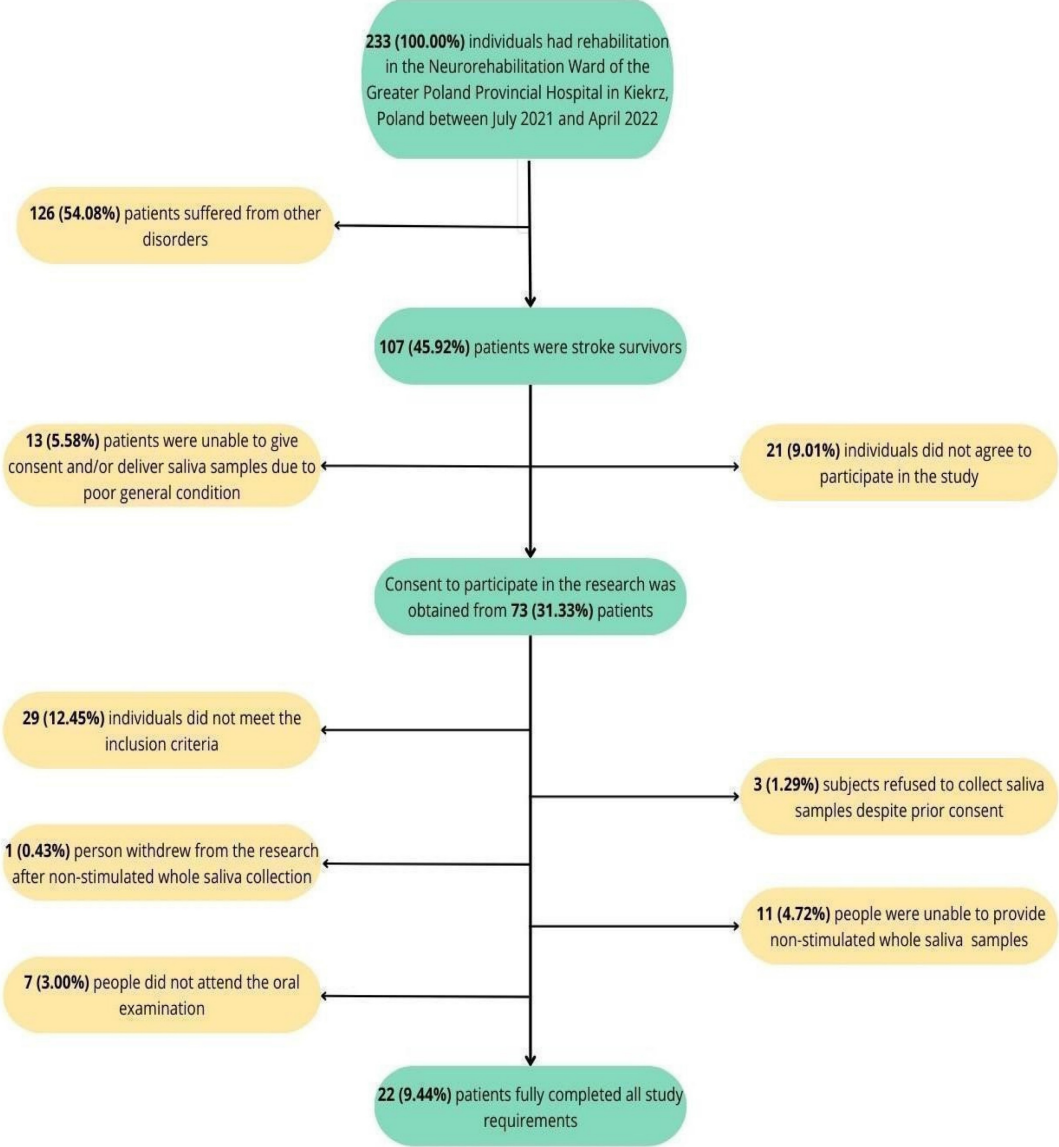
Patient selection flow chart.

Participation in the study was voluntary, and each patient provided written informed consent. A total of 107 stroke patients were initially recruited for the study within the first two days of admission to the ward (between 10-13 days after stroke). Subsequently, 13 participants were excluded due to poor general condition, 21 declined to participate, and 3 individuals, despite signing the consent form, objected to saliva sample collection. After applying the inclusion and exclusion criteria, the number of participants was reduced to 44. One person withdrew from the study after saliva collection. Eleven participants were unable to provide saliva samples, and 7 did not attend the oral examination (Fig 1).

The clinical characteristics of the participants and control group are presented in the supplementary material (Table S1).

### Control Group

The control group included 22 generally healthy individuals - volunteers attending dental check-ups at the Department of Restorative Dentistry of the Medical University of Bialystok (Bialystok, Poland). Participants were matched for age, gender, dental status, periodontal status, and oral hygiene to correspond with the study group. Each participant received a health certificate from a doctor before joining the study. A medical history was taken from each patient, including information on chronic diseases, education, smoking and lifestyle. The control group members followed a regular, balanced diet without any restrictions and received standard recommendations regarding physical activity. Participants over the age of 18, able to give informed consent, as well as collect and provide a saliva sample were included in the study. The exclusion criteria included: being under 18 years old, smoking, inability to provide informed consent, and inability to collect and provide a saliva sample.

### Saliva Collection

Patients were enrolled into the study within the first 2 days after admission to the ward, between 10 and 13 days following the stroke incident. Patients were informed that they should refrain from eating, drinking, oral hygiene procedures, and vigorous physical activity for at least 8 hours prior to sample collection. Unstimulated saliva collection took place in the morning between 8 and 10 a.m., in a quiet and isolated room. Prior to saliva collection, patients had about 5 minutes to acclimate to the room, then they were asked to rinse their mouth twice with distilled water at room temperature to minimize sample contamination. Unstimulated saliva was collected using the spitting method. Patients were instructed to lean forward slightly and spit saliva into a test tube. Saliva was collected into a sterile calibrated tube, which was placed in a cup of ice during the procedure. The duration of saliva collection was 10 minutes. The saliva samples were then centrifuged at the specified parameters: at a temperature of +4 °C, for 20 minutes, 3000×g (MPW 351, MPW Med. Instruments, Warsaw, Poland). The supernatant fluid was preserved for analysis and stored at -80°C for no longer than 3 months 21, 22.

### Oral Examination

An oral examination was performed after the saliva samples were collected. Patients were invited to a separate room, where according to WHO criteria, an artificial light source, a dental mirror, and a probe were used. The oral examination was conducted by a dentist who sat opposite the patient. A dental chart was created for each patient. An assessment was made of the number of teeth with caries (DT), those with fillings due to caries (FT), and extracted teeth due to caries (MT). This information was used to calculate the DMFT index (Decay-Missing-Filled Teeth index), which is the sum of the above components. Oral hygiene was assessed using the Plaque Index (PI) and gingival status with the use of the Gingival Index (GI).

PI was calculated based on the following criteria:

0 - no plaque.

1 - plaque visible only after probing.

2 - moderate plaque visible to the naked eye.

3 - large amount of dental plaque 23.

GI was assessed based on the scale:

0 - no gingival inflammation.

1 - gingival swelling and redness.

2 - gingival swelling, redness, and bleeding on probing.

3 - swelling, redness, and spontaneous gingival bleeding 23.

Before the oral examination, calibration and training of dentists (D.F. and K.G.) were done by another experienced dental specialist (A.Z.). The online Cohen Kappa calculator was used to assess intra-examiner and inter-examiner agreement. The intra- and inter-examiner agreement for PI and GI was assessed in 10 subjects (k > 0.91).

### Salivary Inflammatory Profile

The salivary biomarkers were divided into 5 groups:

- **Pro-inflammatory cytokines**: IL-1α (interleukin 1 alpha), IL-1β (interleukin 1 beta), IL-7 (interleukin 7), IL-16 (interleukin 16), IL-18 (interleukin 18), TNF-α (tumor necrosis factor alpha), TNF-β (tumor necrosis factor beta).
- **Anti-inflammatory cytokines**: IL-1ra (interleukin 1 receptor antagonist), IL-10 (interleukin 10), IL-13 (interleukin 13), TRAIL (tumor necrosis factor-related apoptosis-inducing ligand).
- **Th1 cytokines:** IFN-α2 (interferon-alpha 2), IFN-**γ (**interferon gamma), IL-2 (interleukin 2), IL-2Rα/CD25 (interleukin 2 receptor alpha / cluster of differentiation 25), IL-12 (p40): (interleukin 12 homodimer), IL-12 (p70): (interleukin 12 homodimer), IL-15 (interleukin 15).
- **Th2 cytokines**: IL-4 (interleukin 4), IL-5 (interleukin 5), IL-6 (interleukin 6), IL-9 (interleukin 9).
- **Th17 cytokines**: IL-17 (interleukin 17).

The concentration of salivary cytokines was measured using the Bio-Plex Pro Human Cytokine Assay (Bio-Rad Laboratories, Inc., Hercules, CA, USA). Bio-Plex technology uses magnetic beads to which antibodies directed against the desired biomarkers are attached. Initially, the saliva samples come into contact with the magnetic beads to induce a reaction of antibodies with the marker of interest. This is followed by a series of rinses to remove the unbound proteins. Next, a biotinylated detection antibody is added, forming a sandwich compound, which after the addition of streptavidin-phycoerythrin (SA-PE) conjugate, becomes the final complex. The results are read using a dedicated plate reader kit: the Bio-Plex 200 (Bio-Rad Laboratories, Inc., Hercules, CA, USA). The level of salivary biomarkers was standardized to the content of total protein assessed by the bicinchoninic acid method using the Thermo Scientific PIERCE BCA Protein Assay kit (Rockford, IL, USA).

### Functional Status

The functional status of stroke patients was assessed based on:

- **Addenbrooke's Cognitive Examination Revised** (ACE-R): It is used to determine the cognitive status of patients. Based on the assessment of 5 cognitive domains the patients can receive a total score of 100 points. Higher scores indicate better cognitive abilities 24, 25.
- **Barthel Index** (BI) **of ADL** (activities of daily living): Evaluates patients' ability to perform basic daily activities independently. This scale contains 10 items with different weights measuring the ability to perform daily activities. The maximum score of 20 points indicates complete independence 26.
- **Functional Independence Measure** (FIM): Assesses the amount of assistance a patient needs to perform basic daily activities. The scale includes 18 activities divided into motor and cognitive subgroups. Each activity is rated on a scale of 1 to 7 points. The results show the degree of dependence of patients in performing tasks, with 120-126 points indicating complete independence 27.

**Sitting Balance Scale** (SBS): Evaluates postural balance while sitting. The assessment of balance is performed in 11 situations, each scored from 0 to 4, with higher scores indicating better balance 28.

### Statistical Analysis

Statistical analysis was performed using GraphPad Prism 10 (GraphPad Software, La Jolla, CA, USA) and Past 4.13 (Øyvind Hammer). The Kolmogorov-Smirnov test was used to check the distribution of results. Due to the lack of normal distribution, the Mann-Whitney U test was used for comparisons between two groups. The Spearman rank-order correlation coefficient (Spearman's correlation) was used to check the correlation between salivary markers and clinical data. Receiver Operating Characteristic (ROC) analysis was used to assess the diagnostic utility of salivary cytokines. AUC (area under the curve) and optimal cut-off values were determined for each parameter, ensuring high sensitivity and specificity. The assumed statistical significance was p < 0.05. The size of the study and control groups was determined based on the results of salivary IL-1β and IFN-γ from a pilot study. The ClinCalc online calculator was used, with the power of the statistical test set at 0.8 (α = 0.05). The minimum number of subjects in each group was 18.

## Results

### Pro-inflammatory cytokines

**Figure 2:**
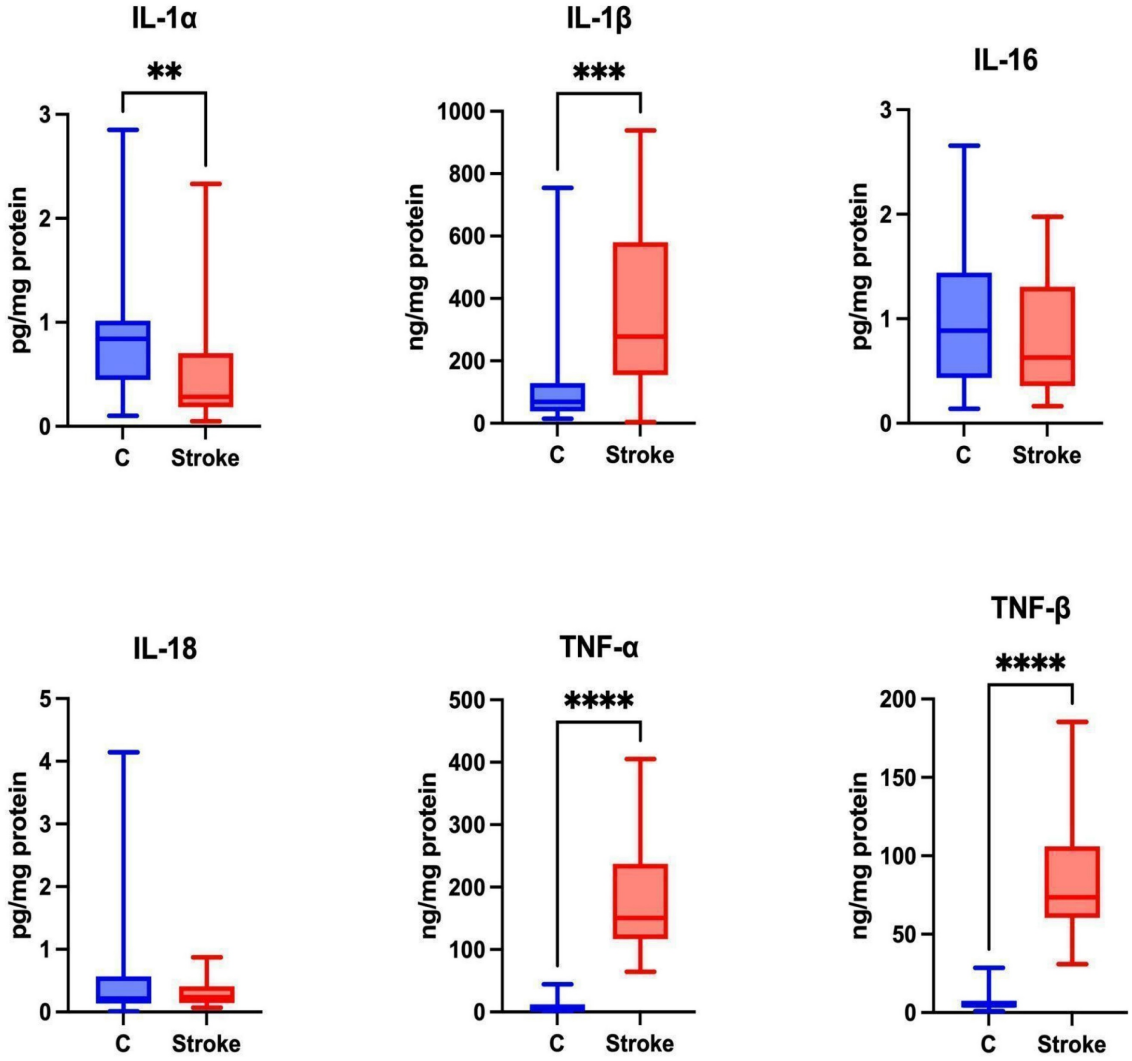
** Content of proinflammatory cytokines in non-stimulated saliva of stroke patients compared to healthy controls.** IL-1α: interleukin 1alpha; IL-1β: interleukin 1beta; IL-16: interleukin 16; IL-18: interleukin 18; TNF-α: tumor necrosis factor alpha; TNF-β: tumor necrosis factor beta; **—p < 0.01; ***—p < 0.001; ****—p < 0.0001.

Pro-inflammatory cytokines trigger brain inflammation 29, influencing the severity of stroke and leading to worse mental and physical functioning in stroke patients 30. Statistically higher levels of IL-1β, TNF-α and TNF-β were observed in the unstimulated saliva of the study group compared to the control group (p=0.0003; p ≤ 0.0001; p ≤ 0.0001, respectively). A significantly lower level of IL-1α was observed in the unstimulated saliva of the study group compared to the control group (p = 0.0024). No statistically significant differences were found between the study and control groups for IL-16 (p = 0.51) and IL-18 (p = 0.7361) (Figure 2).

The concentration of IL-7 in unstimulated saliva was below the level of detection.

### Anti-inflammatory Cytokines

**Figure 3:**
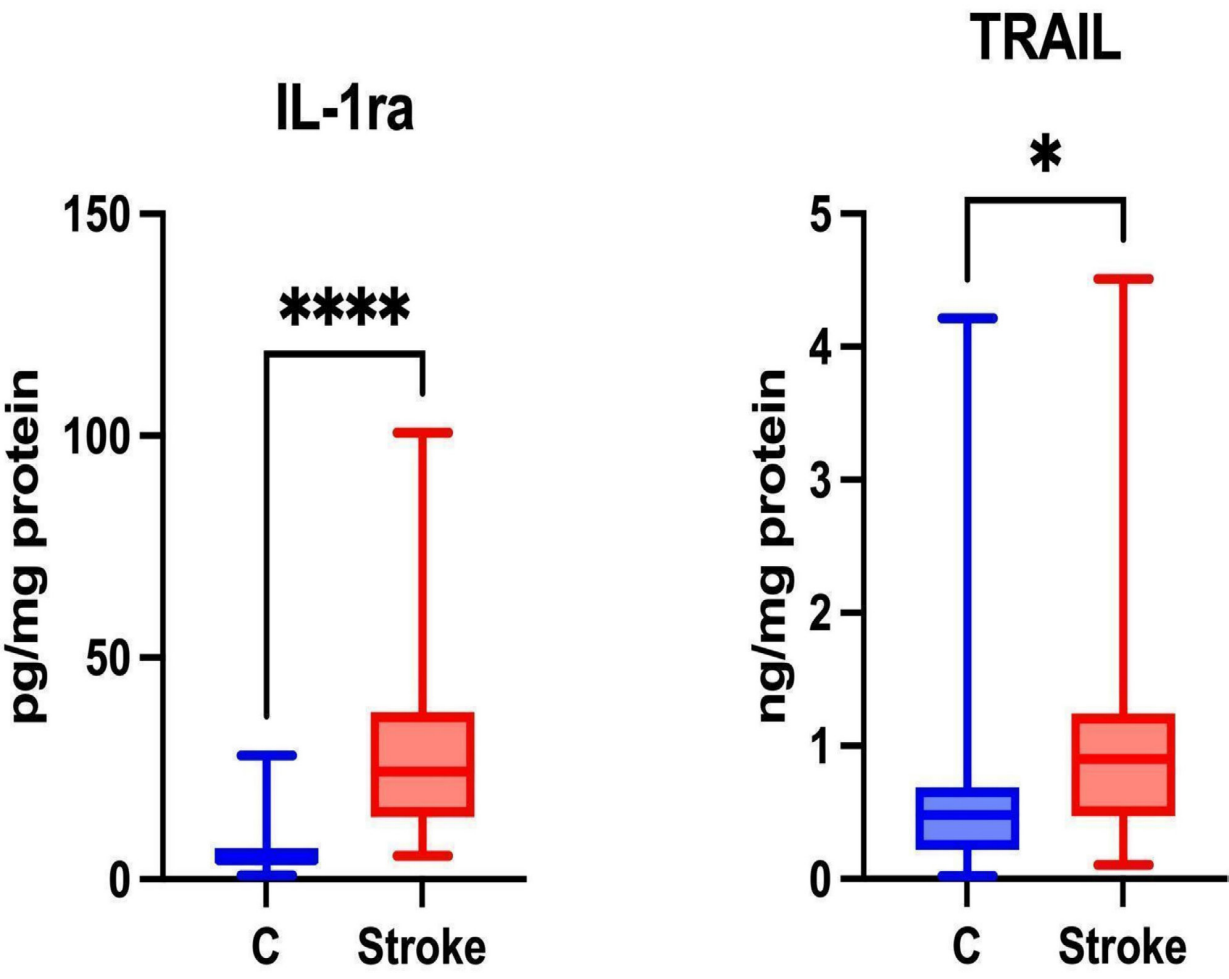
** Content of anti-inflammatory cytokines in non-stimulated saliva of stroke patients compared to healthy controls.** IL-1ra: interleukin 1 receptor antagonist; TRAIL: tumor necrosis factor-related apoptosis-inducing ligand; *—p < 0.05; ****—p < 0.0001.

The expression of anti-inflammatory cytokines is driven by their role in modulating the immune response, either through cytokine receptors or by involving inhibitors that regulate inflammation 31. Anti-inflammatory therapies help reduce brain damage resulting from ischemia 32. Significantly higher levels of IL-1ra and TRAIL were noted in the unstimulated saliva of patients in the study group compared to the control group (p ≤ 0.0001; p = 0.0206; respectively) (Figure 3).

The concentrations of IL-10 and IL-13 in saliva were below the level of detection.

### Th1 Cytokines

**Figure 4:**
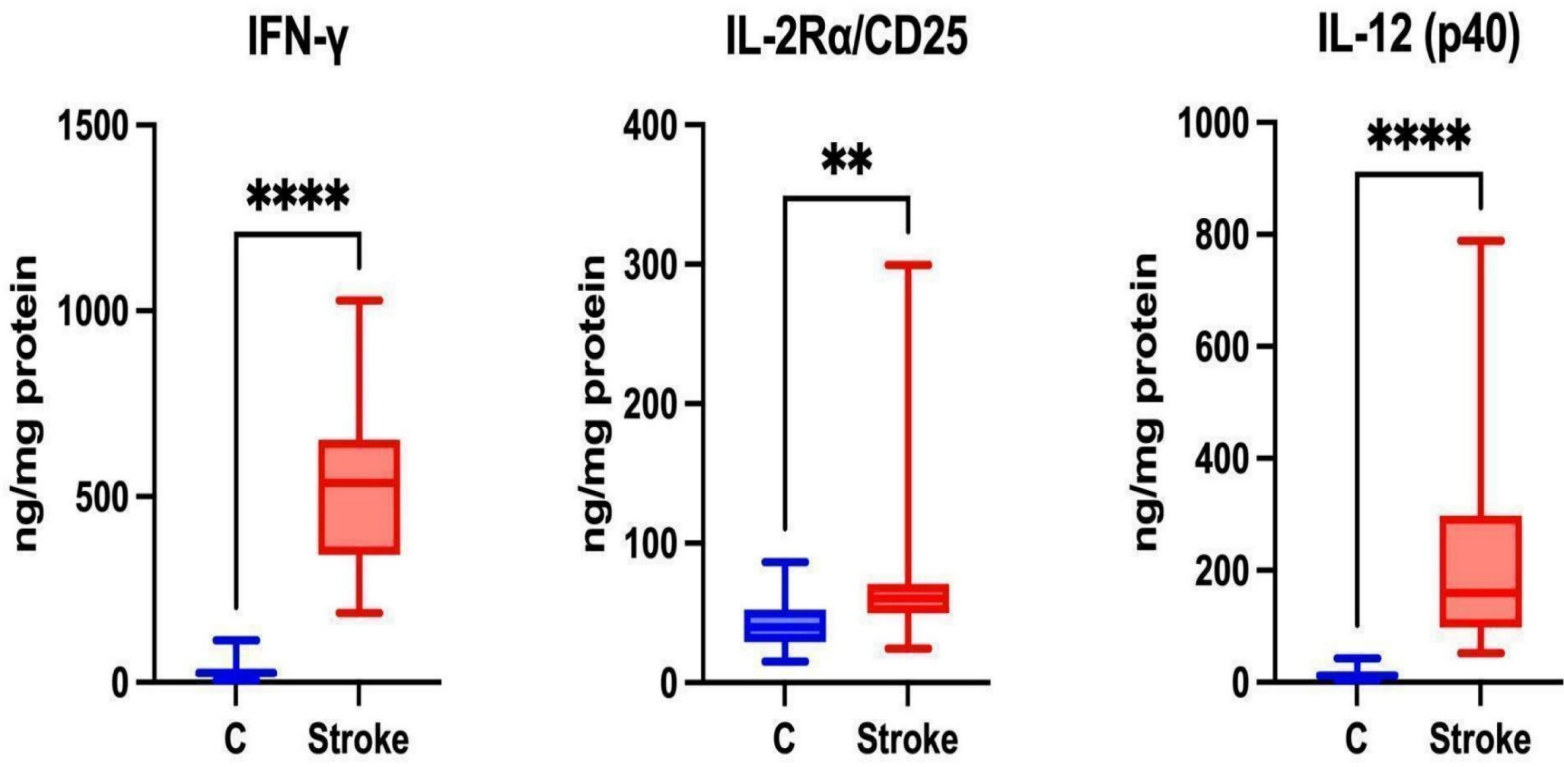
** Content of Th1 cytokines in non-stimulated saliva of stroke patients compared to healthy controls.** IFN-**γ**: interferon gamma; IL-2Rα/CD25: interleukin 2 receptor alpha/cluster of differentiation 25; IL-12 (p40): interleukin 12 homodimer; **—p < 0.01; ****—p < 0.0001.

Th1 cells are associated with the secretion of pro-inflammatory Th1 cytokines such as IFN-γ, IL-2Rα/CD25, and IL-12 33. Th1 lymphocytes participate in the cellular immune response 34. Statistically higher levels of IFN-γ, IL-2Rα/CD25 and IL-12 (p40) were noted in the unstimulated saliva of patients in the study group compared to the control group (p ≤ 0.0001; p=0.0021; p ≤ 0.0001, respectively) (Figure 4).

The concentrations of IFN-α2, IL-2, IL-12 (p70), and IL-15 were below the level of detection.

### Th2 Cytokines

**Figure 5:**
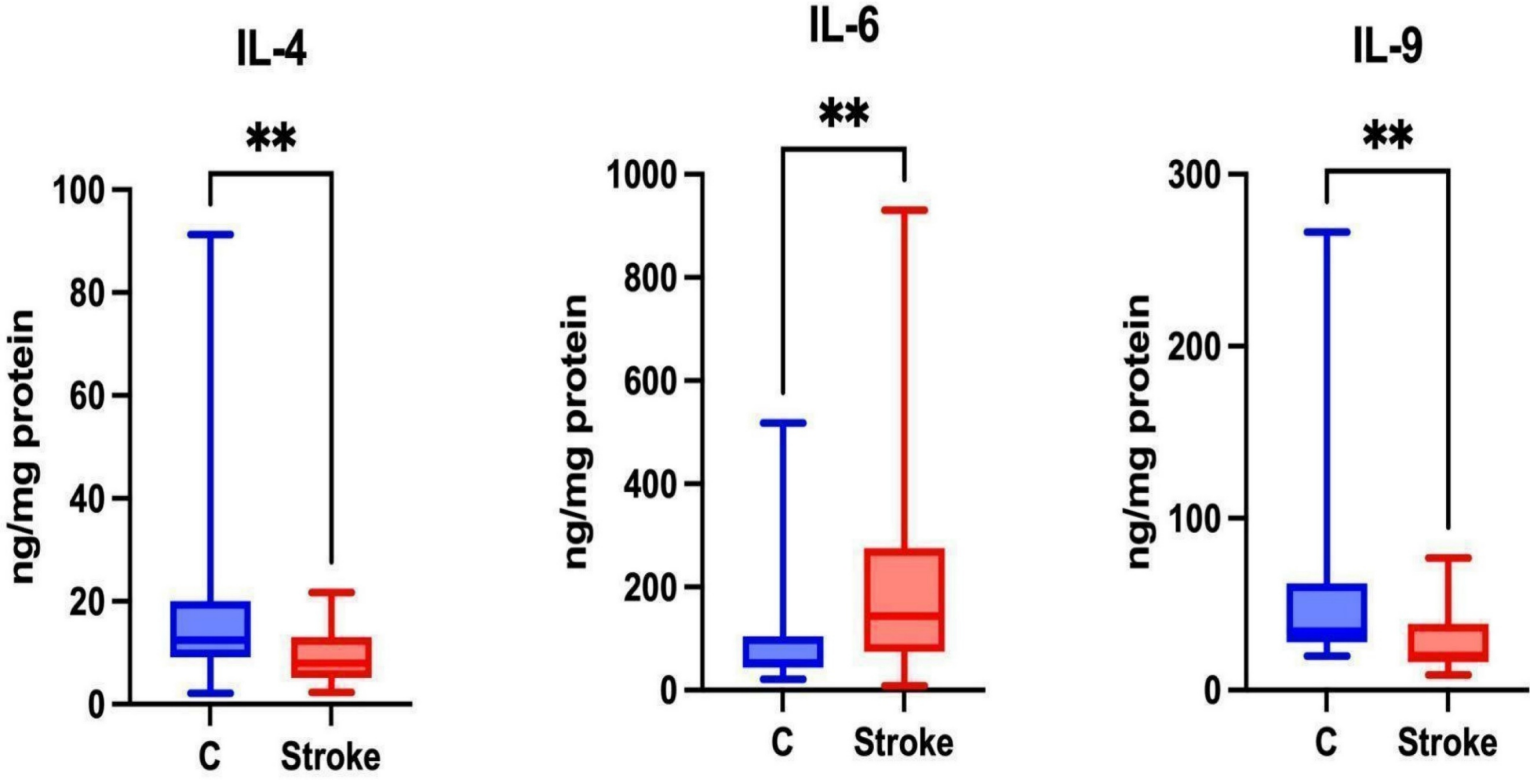
Content of Th2 cytokines in non-stimulated saliva of stroke patients compared to healthy controls. IL-4: interleukin 4; IL-6: interleukin 6; IL-9: interleukin 9; **—p < 0.01.

Th2 cells are responsible for the secretion of Th2 cytokines such as IL-4, IL-6, and IL-9 35. Th2 lymphocytes are associated with the humoral immune response 34. Significantly higher levels of IL-6 were noted in the unstimulated saliva of patients in the study group compared to the control group (p = 0.0023). Significantly lower levels of IL-4 and IL-9 were also noted in the unstimulated saliva of patients in the study group compared to the control group (p = 0.0059; p = 0.0059, respectively) (Figure 5).

The concentration of IL-5 in unstimulated saliva was undetectable.

### Th17 Cytokines

Th17 cells are associated with autoimmune diseases 36, and during a stroke, they have the ability to secrete pro-inflammatory Th17 cytokines that exacerbate neuroinflammation 33.

**Figure 6:**
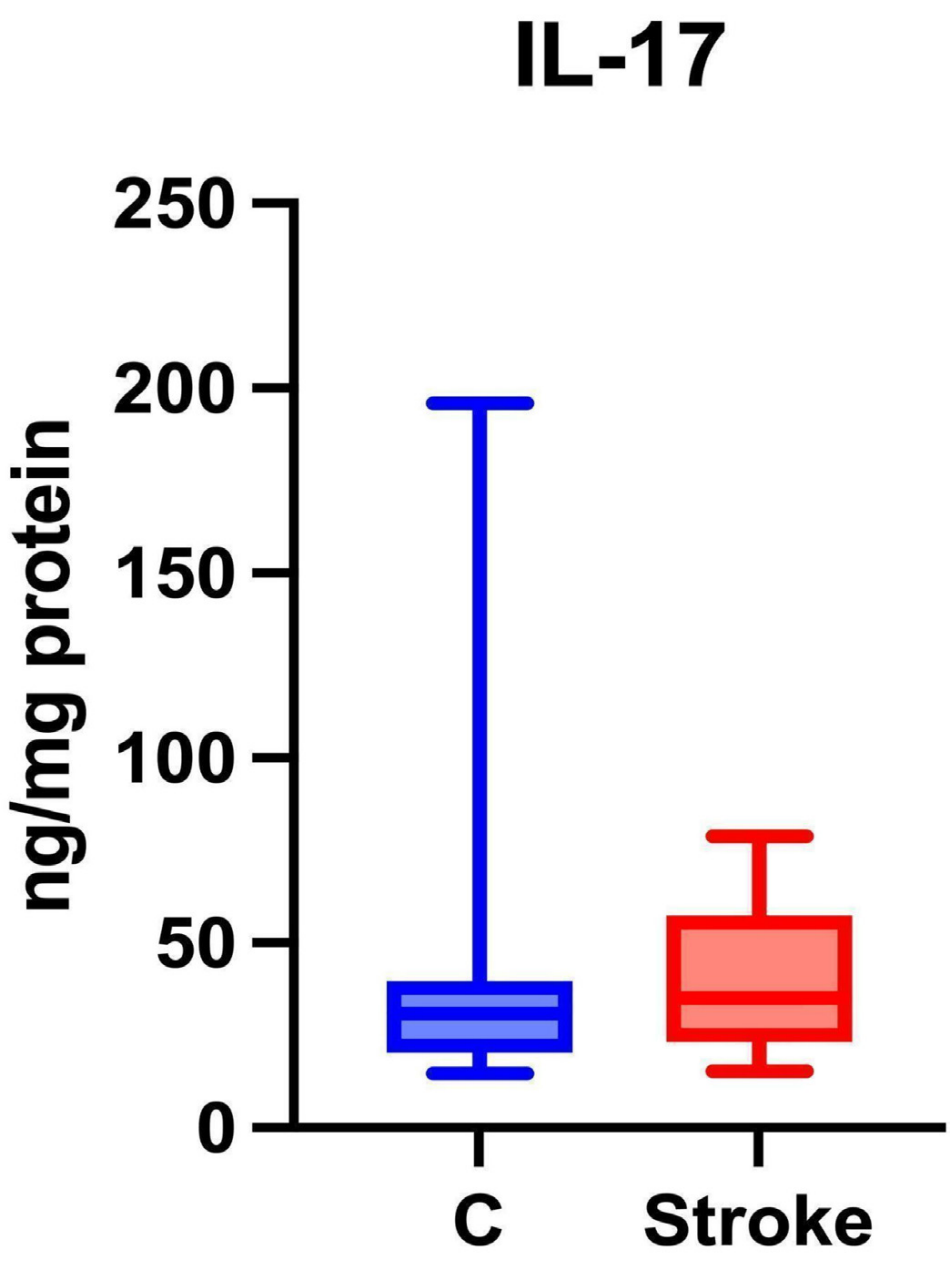
Content of Th17 cytokines in non-stimulated saliva of stroke patients compared to healthy controls. IL-17: interleukin 17.

No statistically significant differences were found between the study group and the control group regarding salivary IL-17 levels (p = 0.4219) (Figure 6).

### Correlations

**Figure 7:**
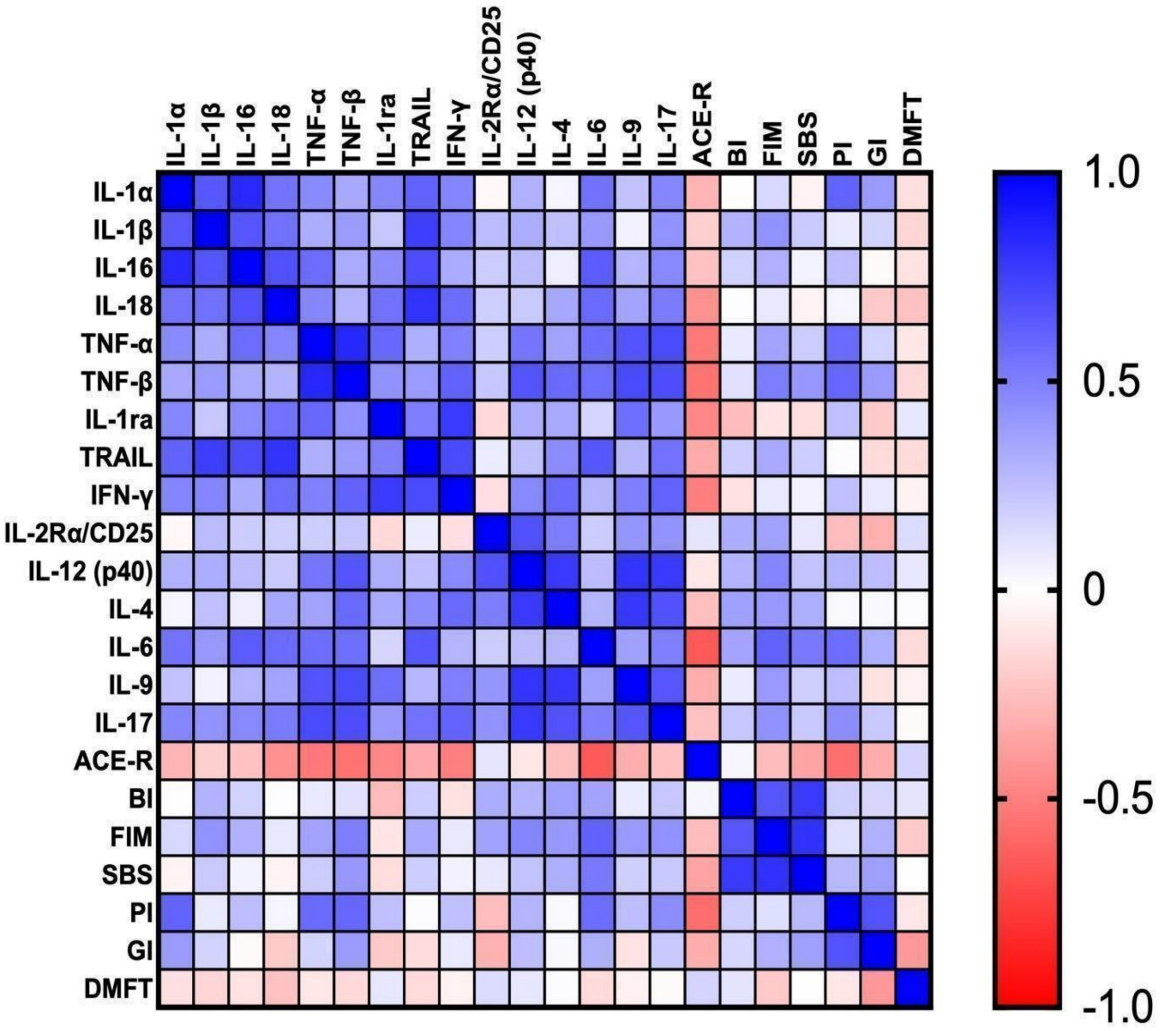
** Correlations between the studied parameters.** ACE-R: Addenbrooke's Cognitive Examination Revised; BI: Barthel Index; DMFT: The Decayed, Missing and Filled Teeth; GI: Gingival Index; FIM: The Functional Independence Measure; IL-1α: interleukin 1alpha; IL-1β: interleukin 1beta; IL-1ra: interleukin 1 receptor antagonist; IL-2Rα/CD25: interleukin 2 receptor alpha/cluster of differentiation 25; IL-4: interleukin 4; IL-6: interleukin 6; IL-9: interleukin 9; IL-12 (p40): interleukin 12 homodimer; IL-16: interleukin 16; IL-17: interleukin 17; IL-18: interleukin 18; IFN-**γ**: interferon gamma; PI: Plaque Index; SBS: Sitting Balance Scale; TNF-α: tumor necrosis factor alpha; TNF-β: tumor necrosis factor beta; TRAIL: tumor necrosis factor-related apoptosis-inducing ligand.

Correlations between the parameters are presented in Figure 7.

Salivary IL-1α correlated positively with IL1β (p = 0.001, r = 0.66) and TRAIL (p = 0.004, r = 0.61). We observed positive correlations between IL-1β and IL-16 (p = 0.001, r = 0.66). IL-16 correlated positively with IL-18 (p = 0.001, r = 0.68) and IL-6 (p = 0.002, r = 0.64). Positive correlation was also noted between TNF-α and IL-9 (p = 0.001, r = 0.67). TNF-β correlated positively with IFN-γ (p = 0.005, r = 0.61), IL-12 (p = 0.002, r = 0.67), IL-9 (p = 0.001, r = 0.71) and IL-17 (p = 0.001, r = 0.7). Moreover, TRAIL was positively associated with IL-6 (p = 0.002, r = 0.66), whereas the positive association was observed between IFN-γ and IL-17 (p = 0.003; r = 0.61). We observed positive correlation between IL-2Rα and IL-12 (p = 0.001; r = 0.68). IL-17 presented a positive correlation with IL-4 (p = 0.001, r = 0.68) and IL-9 (p = 0.002; r = 0.66).

IL-1α presented a correlation with IL-18 (p = 0.01, r = 0.55), TNF-α (p = 0.04, r = 0.46), IL-1ra (p = 0.04, r = 0.47), IFN-γ (p = 0.03, r = 0.48), IL-6 (p = 0.01, r = 0.55) and IL-17 (p = 0.03; r = 0.47). IL-1β presented an association with IL-18 (p = 0.01, r = 0.56) and IFN-γ (p = 0.03, r = 0.48). IL-16 presented a correlation with TNF-α (p = 0.01, r = 0.57), IL-1ra (p = 0.04, r = 0.45) and IL-17 (p = 0.03, r = 0.46). IL-18 correlated with TNF-α (p = 0.03, r = 0.47), IL-1ra (p = 0.01, r = 0.55), IFN-γ (p = 0.01, r = 0.57), IL-6 (p = 0.01, r = 0.58) and IL-17 (p = 0.01, r = 0.52). TNF-α correlated with IL-1ra (p = 0.01, r = 0.59), IFN-γ (p = 0.02, r = 0.49), IL-12 (p = 0.01, r = 0.54) and IL-6 (p = 0.01, r = 0.57). TNF-β was associated with IL-4 (p = 0.01; r = 0.58) and IL-6 (p = 0.01, r = 0.57). IL-1ra presented a correlation with TRAIL (p = 0.02, r = 0.51) and IL-9 (p = 0.01, r = 0.56). Moreover, TRAIL was associated with IL-4 (p = 0.047, r = 0.45) and IL-17 (p = 0.01, r = 0.55). IFN-γ presented an association with IL-12 (p = 0.04, r = 0.47), IL-4 (p = 0.01, r = 0.58) and IL-9 (p = 0.03, r = 0.495). There was a correlation between IL-2Rα and IL-4 (p = 0.02, r = 0.51). The correlation was also noted between IL-6 and IL-17 (p = 0.02, r = 0.50).

We found a negative correlation between ACE-R and salivary TNF-α (p = 0.01, r = -0.53), TNF-β (p = 0.02, r = -0.60) and IFN-γ (p = 0.02, r = -0.50). On the other hand, FIM presented a positive association with IL-6 (p = 0.003, r = 0.62) and BI (p = 0.001, r = 0.66). The positive correlation was also noted between SBS and IL-6 (p = 0.02, r = 0.52). PI correlated positively with IL-1α (p = 0.02, r = 0.61), TNF-α (p = 0.03, r = 0.58), TNF-β (p = 0.03, r = 0.6), IL-6 (p = 0.04, r = 0.57) and ACE-R (p = 0.03, r = -0.57). Interestingly, GI correlated positively only with PI (p = 0.01, r = 0.67).

A significant correlation was noted between ACE-R and IL-18 (p = 0.04; r = -0.43), IL-1ra (p = 0.03, r = -0.47) and IL-6 (p = 0.001, r = -0.25). Furthermore, FIM was associated with TNF-β (p = 0.027, r = 0.51), IL-12 (p = 0.03, r = 0.48) and IL-17 (p = 0.048, r = 0.43).

### ROC Analysis

In our study, the salivary cytokine levels significantly distinguish ischemic stroke patients from healthy individuals. Among the analyzed markers, salivary TNF-α, TNF-β, IFN-γ, and IL-12 demonstrated exceptional diagnostic accuracy, attaining an AUC of 1 with 100% sensitivity and specificity (Table 1).

## Discussion

In our previous study, we evaluated the diagnostic potential of unstimulated and stimulated saliva in assessing TNF-α, IL-6 and IL-10 in stroke patients 16. Since unstimulated saliva proved more useful, this study focused on a detailed evaluation of its inflammatory profile. We found significantly higher levels of salivary pro-inflammatory cytokines (IL-1β, TNF-α, TNF-β), anti-inflammatory cytokines (IL-1ra, TRAIL), Th1 cytokines (IFN-γ and IL-12 (p40)), and Th2 cytokines (IL-6) in patients with ischemic stroke compared to healthy participants. However, the levels of IL-1α, IL-4, and IL-9 were significantly lower in the saliva of the study group. The obtained results do not indicate a dominance of any particular branch of the immune response.

Stroke is one of the leading causes of death worldwide. With the rising prevalence of stroke, developing simple and effective diagnostic methods to monitor disease progression is essential. Recently, saliva has gained increasing attention due to its non-invasive nature, safety, and the ability to collect multiple samples without requiring specialized personnel. The use of stress-free biofluid collection minimizes patient discomfort, which is especially important for the elderly. Whole saliva is a mixture of salivary gland filtrate, gingival fluid, nasal and pharyngeal secretions, cells lining the oral cavity, and microorganisms 37. Moreover, due to the abundant vascularization of the salivary glands, a significant portion of the molecules that are present in blood can enter the saliva 38. Cytokines can move from blood to saliva through active transport, ultrafiltration, or passive diffusion 39, 40. Additionally, cytokines can enter the saliva through gingival fluid, transudation across the oral mucosa, and inflamed areas where "leaky patches" develop 41. The dental examinations revealed that stroke patients had poor oral hygiene and periodontal health, which could make leaky patches a major source of cytokines in saliva. Moreover, the levels of salivary cytokines positively correlated with dental indices. We found a particularly strong relationship between pro-inflammatory cytokines (IL-1α, TNF-α, TNF-β, IL-6) and the Plaque Index (PI), and between IL-1α and TNF-β and the Gingival Index (GI). It is well known that dental caries and periodontal diseases significantly increase the risk of stroke 42. The presence of even one tooth with caries correlates with an increased risk of stroke 43. Dental infections starting within the tooth or in the periodontium can spread to nearby bones and soft tissues 44. Periodontal dysbiosis not only triggers a local inflammatory response but also stimulates systemic inflammation through the dissemination of pathogens in the bloodstream 45. Systemic inflammation, molecular mimicry, and bacteremia can lead to atherosclerosis and cardiovascular diseases 46-48. Bacteria associated with periodontitis, such as *Porphyromonas gingivalis* and *Prevotella intermedia*, can disrupt the endothelial barrier and thus enter the atherosclerotic plaques directly from the bloodstream 49. Therefore, moderate and severe periodontitis contribute to the progression of atherosclerosis, which in turn promotes the development of stroke 50. Since oral inflammation is the primary source of salivary cytokines 51, 52, our study included a control group of individuals with comparable oral hygiene and periodontal health. This allowed for proper comparison of results between the study and control groups.

Inflammation resulting from a stroke is associated with secondary damage to brain tissues. During a stroke, the balance between pro-inflammatory and anti-inflammatory molecules is disturbed 29. TNF-α, as one of the main pro-inflammatory cytokines, plays a significant role in enhancing the inflammatory response by regulating the expression of adhesion molecules and inducing the synthesis of free radicals 53. In our study, the content of salivary TNF-α was significantly higher in stroke patients and correlated with cognitive impairment on the ACE-R scale. The ACE-R scale is used for initial differential diagnosis of dementia syndromes and for monitoring of disease progression. This scale assesses attention, orientation, memory, verbal fluency, language and visuospatial functions. It has been shown that ACE-R can be used for early assessment of patients with cerebrovascular changes 54. We observed similar correlations in the ACE-R scale for TNF-β and IL-6. TNF-β activates signaling pathways associated with the transcription factor NF-κB (nuclear factor kappa-light-chain-enhancer of activated B cells) and MAPK (mitogen-activated protein kinase), thereby stimulating inflammation and cell death leading to cognitive impairment 55, 56. IL-6 increases prothrombotic activity and activates endothelial cells 57. During the inflammatory process, IL-6 activates signaling pathways responsible for oxidative stress, proliferation, and lymphocyte differentiation 53. However, IL-6 may have dual effects in brain tissue - in the early stage of stroke it shows pro-inflammatory activity, while in later stages, it has a potentially neurotrophic effect 4. The protective effect of IL-6 may be indicated by the positive correlation of salivary IL-6 with the functional status of patients on the FIM and SBS scales.

The concentration of Th1 cytokines (IFN-γ and IL-12) and anti-inflammatory cytokines (IL-1ra, TRAIL) were also significantly higher in the saliva of stroke patients. IFN-γ synthesized by Th1 cells stimulates the polarization of microglia into the M1 phenotype, which exhibits pro-inflammatory activity, and activates the JAKs (Janus kinases) and STAT (signal transducer and activator of transcription proteins) signaling pathways 4. It also exerts pro-inflammatory and pro-thrombotic effects by regulating adhesion molecule expression, activating the NADPH oxidase complex, and inducting adhesion molecule synthesis 58. On the other hand, IL-12 stimulates the production of pro-inflammatory cytokines and chemotactic proteins, while also inducing the expression of adhesion molecules on endothelial cells to facilitate the influx of blood cells 59. Anti-inflammatory cytokines modulate the immune response through various molecular mechanisms 60. Interestingly, the concentration of Th2 cytokines in saliva (except for pro-inflammatory IL-6) was significantly lower in the study group compared to the control group. The differentiation of helper lymphocytes into Th2 is promoted by interleukins IL-4 and IL-9, and their reduced expression may indicate an impaired humoral response in stroke patients.

In our study, the salivary concentration of IL-2, IL-5, IL-7, IL-10, IL-12 (p70), IL-13, IL-15, and IFN-α2 was below the detection level in healthy controls (most commonly) and in stroke patients. Therefore, we cannot fully characterize the impact of stroke on the salivary cytokine profile. Indeed, the low concentration of certain biomarkers in saliva poses a limitation to its use in laboratory medicine 61. The level of salivary biomarkers is also influenced by local factors (caries and periodontal inflammation 38) and general factors (body hydration level, olfactory stimulation, time of day (during saliva collection), or time since the last meal 62). However, saliva collection has numerous advantages. It is a non-invasive, non-infectious, simple, and inexpensive procedure 63 that is comfortable for patients of all ages 64. Unlike blood, saliva can be safely collected from individuals with coagulation disorders without the involvement of medical personnel 65.

According to the Biomarkers Definition Working Group, a biomarker is a characteristic that is objectively measured and evaluated as an indicator of normal biological processes, pathological processes, or pharmacological responses to a therapeutic intervention 66. Currently, there is no widely available and specific biomarker for stroke diagnosis. This prevents early diagnosis of the disease and above all, effective treatment of patients. An ideal stroke biomarker should have high sensitivity and specificity, be easily measurable in circulating biological fluids, and provide information on the effectiveness of treatment. More and more studies assess the biomarker potential of cytokines in stroke diagnosis. Christensen et al. assessed the levels of TNF-α, IL-1, IL-1ra, and IL-10 in the blood of 179 stroke patients. At the onset of stroke, the levels of these cytokines were significantly higher than three months after the incident 67. In our study, we also noted higher levels of TNF-α, IL-1, and IL-1ra in the study group, however, IL-10 was undetectable in saliva. Beamer et al. showed that serum IL-Ra and IL-6 levels were significantly higher in stroke patients compared to healthy individuals 68. Kim et al., in addition to elevated IL-6 levels, also showed a significant increase in IL-4 levels, and a considerable decrease in IL-2 levels, in the blood of stroke patients 69. Tan et al. observed significantly higher levels of IL-4 and IL-9 in the blood of stroke patients, while IFN-γ levels did not differ between the groups 70. ROC diagnostic utility analysis in our study indicates that salivary TNF-α, TNF-β, IFN-γ, and IL-12 levels significantly differentiate ischemic stroke patients from healthy individuals (AUC = 1, sensitivity = 100%, specificity = 100%). Although the number of patients in our study was statistically calculated, verification of results on a larger population of patients is necessary. Salivary cytokines may be a potential diagnostic biomarker for stroke. Saliva is particularly attractive as a diagnostic material for brain diseases 71, 72. For many biomarkers, a stronger brain-saliva correlation has been demonstrated than a brain-blood correlation, which may be particularly important in the diagnosis of stroke 73-75. Unfortunately, we did not assess the inflammatory profile of the blood of stroke patients, so we cannot compare inflammatory changes at the systemic (blood) and local (saliva) level. However, many studies indicate a strong saliva-blood correlation for cytokines 76-78.

### Limitations

Our study also has numerous limitations. One of the biggest is that we only analyzed the inflammatory profile of unstimulated saliva. Saliva composition may differ depending on its type - unstimulated saliva is primarily produced by the submandibular glands (constituting plasma filtrate), while stimulated saliva mainly comes from the parotid glands and is less sensitive to systemic factors 79-82. Furthermore, since we did not analyze the inflammatory profile in the blood and cerebrospinal fluid, we cannot determine whether the inflammation originates locally (from the salivary glands) or centrally (from the brain). Therefore, further analyses comparing different circulating fluids in a larger stroke patient population are necessary. Additionally, a comparison of the salivary inflammatory profiles between patients with ischemic and hemorrhagic stroke should also be explored.

## Conclusions

**Figure 8:**
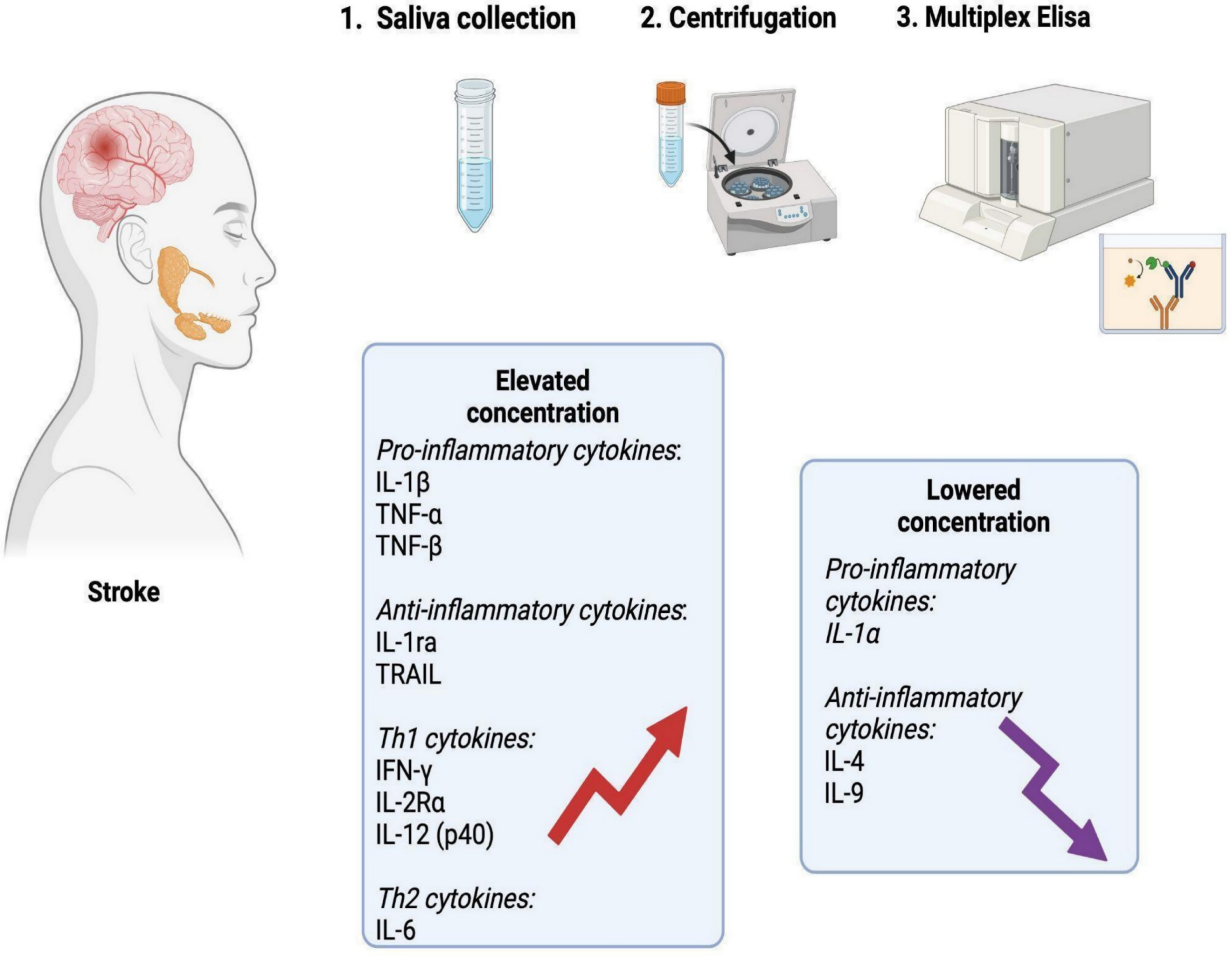
** Graphical conclusions of the study (generated using biorender.com).** In the unstimulated saliva of stroke patients, a significantly higher content of pro-inflammatory cytokines (IL-1β, TNF-α, TNF-β), anti-inflammatory cytokines (IL-1ra, TRAIL), Th1 cytokines (IFN-**γ**, IL-2Rα and IL-12 (p40)) and Th2 cytokines (IL-6) was found compared to healthy individuals. The content of IL-1α, IL-4 and IL-9 was significantly lower in the saliva of patients from the study group. IL-1α: interleukin 1alpha; IL-1β: interleukin 1beta; IL-1ra: interleukin 1 receptor antagonist; IL-2Rα: interleukin 2 receptor alpha; IL-4: interleukin 4; IL-6: interleukin 6; IL-9: interleukin 9; IL-12 (p40): interleukin 12 homodimer; IFN-**γ**: interferon gamma; TNF-α: tumor necrosis factor alpha; TNF-β: tumor necrosis factor beta; TRAIL: tumor necrosis factor-related apoptosis-inducing ligand.

In summary, stroke patients exhibit changes in salivary composition including an increased secretion of inflammatory mediators. The obtained results confirm the inflammatory etiology of ischemic stroke, but do not indicate the dominance of any branch of the immune response. The levels of salivary TNF-α, TNF-β, IFN-γ, and IL-12 significantly differentiate ischemic stroke patients from healthy individuals, making salivary cytokines a potential biomarker for stroke (Figure 8). Further studies examining cytokine levels in the saliva and blood of a larger stroke patient population are necessary.

## Key messages

Stroke patients exhibited modified salivary composition, with an increased release of inflammatory markers.

TNF-α, TNF-β, IFN-γ, and IL-12 are markers, which are useful to distinguish ischemic stroke survivors from healthy controls.

While it is essential to validate these results in a larger patient population, salivary cytokines could serve as virtual diagnostic markers.

## Supplementary Material

Supplementary table S1.

## Figures and Tables

**Table 1:** ** Receiver Operating Characteristic (ROC) analysis for salivary cytokines.** IL-1alpha: interleukin 1alpha; IL-1beta: interleukin 1beta; IL-1ra: interleukin 1 receptor antagonist; IL-2Ralpha/CD25: interleukin 2 receptor alpha/cluster of differentiation 25; IL-4: interleukin 4; IL-6: interleukin 6; IL-9: interleukin 9; IL-12 (p40): interleukin 12 homodimer; IL-16: interleukin 16; IL-17: interleukin 17; IL-18: interleukin 18; IFN-gamma: interferon gamma; TNF-alpha: Tumor necrosis factor alpha; TNF-beta: Tumor necrosis factor beta; TRAIL: Tumor necrosis factor-related apoptosis-inducing ligand.

Biomarker	Area	Cut off point	Sensitivity %	95% CI	Specificity%	95% CI	Likelihood ratio
*pro-inflammatory cytokines*							
IL-1alpha	0.77	< 0.6638	71.43	50.04 - 86.19%	61.9	40.88 - 79.25%	1.88
IL-1beta	0.81	> 152.9	81.82	61.48 - 92.69%	81.82	61.48 - 92.69%	4.5
IL-16	0.5599	< 0.7127	59.09	38.73 - 76.74%	59.09	38.73 - 76.74%	1.444
IL-18	0.53	< 0.2655	63.64	42.95 - 80.27%	45.45	26.92 - 65.34%	1.17
TNF-alpha	1	> 21.18	100	83.18 - 100.0%	95.45	78.20 - 99.77%	22
TNF-beta	1	> 21.18	100	83.18 - 100.0%	95.45	78.20 - 99.77%	22
*anti-inflammatory cytokines*							
IL-1ra	0.94	> 6.535	95.24	77.33 - 99.76%	72.73	51.85 - 86.85%	3.49
TRAIL	0.71	> 0.5253	66.67	45.37 - 82.81%	59.09	38.73 - 76.74%	1.63
*Th1 cytokines*							
IFN-gamma	1	> 81.51	100	84.54 - 100.0%	95.45	78.20 - 99.77%	22
IL-2Ralpha	0.77	> 51.58	77.27	56.56 - 89.88%	77.27	56.56 - 89.88%	3.4
IL-12 (p40)	1	> 33.27	100	84.54 - 100.0%	95.45	78.20 - 99.77%	22
*Th2 cytokines*							
IL-4	0.74	< 10.19	76.19	54.91 - 89.37%	59.09	38.73 - 76.74%	1.86
IL-6	0.77	> 64.56	85.71	65.36 - 95.02%	68.18	47.32 - 83.64%	2.69
IL-9	0.75	< 29.90	70	48.10 - 85.45%	63.64	42.95 - 80.27%	1.93
*Th17 cytokines*							
IL-17	0.57	> 32.60	59.09	38.73 - 76.74%	54.55	34.66 - 73.08%	1.3

## References

[1] Ding S, Chen Q, Chen H (2021). The Neuroprotective Role of Neuroserpin in Ischemic and Hemorrhagic Stroke. Curr Neuropharmacol.

[2] Sudharsanan N, Deshmukh M, Kalkonde Y (2019). Direct estimates of disability-adjusted life years lost due to stroke: a cross-sectional observational study in a demographic surveillance site in rural Gadchiroli, India. BMJ Open.

[3] Farina M, Vieira LE, Buttari B (2021). The Nrf2 Pathway in Ischemic Stroke: A Review. Molecules.

[4] Maida CD, Norrito RL, Daidone M (2020). Neuroinflammatory Mechanisms in Ischemic Stroke: Focus on Cardioembolic Stroke, Background, and Therapeutic Approaches. Int J Mol Sci.

[5] Kawabori M, Shichinohe H, Kuroda S (2020). Clinical Trials of Stem Cell Therapy for Cerebral Ischemic Stroke. Int J Mol Sci.

[6] He J, Liu J, Huang Y (2021). Oxidative Stress, Inflammation, and Autophagy: Potential Targets of Mesenchymal Stem Cells-Based Therapies in Ischemic Stroke. Front Neurosci.

[7] Ren X, Hu H, Farooqi I (2021). Blood substitution therapy rescues the brain of mice from ischemic damage. Nat Commun. 2020;25;11(1):4078. doi: 10.1038/s41467-020-17930-x. Erratum in: Nat Commun.

[8] Muhammad S, Barakat W, Stoyanov S (2008). The HMGB1 receptor RAGE mediates ischemic brain damage. J Neurosci.

[9] Fumagalli S, Perego C, Pischiutta F (2015). The ischemic environment drives microglia and macrophage function. Front Neurol.

[10] Liesz A, Dalpke A, Mracsko E (2019). DAMP signaling is a key pathway inducing immune modulation after brain injury. J Neurosci. 2015;14;35(2):583-598. doi: 10.1523/JNEUROSCI.2439-14.2015. Erratum in: J Neurosci.

[11] Simats A, García-Berrocoso T, Montaner J (2016). Neuroinflammatory biomarkers: From stroke diagnosis and prognosis to therapy. Biochim Biophys Acta.

[12] Okada T, Suzuki H, Travis ZD (2020). The Stroke-Induced Blood-Brain Barrier Disruption: Current Progress of Inspection Technique, Mechanism, and Therapeutic Target. Curr Neuropharmacol.

[13] Li W, Pan R, Qi Z (2018). Current progress in searching for clinically useful biomarkers of blood-brain barrier damage following cerebral ischemia. Brain Circ.

[14] Kułak-Bejda A, Waszkiewicz N, Bejda G (2019). Diagnostic Value of Salivary Markers in Neuropsychiatric Disorders. Dis Markers.

[15] Celec P, Tóthová Ľ, Šebeková K (2015). Salivary markers of kidney function - Potentials and limitations. Clin Chim Acta. 2016;30;453:28-37. doi: 10.1016/j. cca.

[16] Maciejczyk M, Mil KM, Gerreth P (2021). Salivary cytokine profile in patients with ischemic stroke. Sci Rep.

[17] Maciejczyk M, Nesterowicz M, Zalewska A (2022). Salivary Xanthine Oxidase as a Potential Biomarker in Stroke Diagnostics. Front Immunol.

[18] Easton JD, Saver JL, Albers GW (2009). Definition and evaluation of transient ischemic attack: a scientific statement for healthcare professionals from the American Heart Association/American Stroke Association Stroke Council; Council on Cardiovascular Surgery and Anesthesia; Council on Cardiovascular Radiology and Intervention; Council on Cardiovascular Nursing; and the Interdisciplinary Council on Peripheral Vascular Disease. The American Academy of Neurology affirms the value of this statement as an educational tool for neurologists. Stroke.

[19] Sacco RL, Kasner SE, Broderick JP (2013). An updated definition of stroke for the 21st century: a statement for healthcare professionals from the American Heart Association/American Stroke Association. Stroke.

[20] Albers GW, Caplan LR, Easton JD (2002). Transient ischemic attack - proposal for a new definition. N Engl J Med.

[21] Szulimowska J, Zalewska A, Taranta-Janusz K (2023). Association Between Salivary Cytokines, Chemokines and Growth Factors and Salivary Gland Function in Children with Chronic Kidney Disease. J Inflamm Res.

[22] Klimiuk A, Zalewska A, Knapp M (2022). Could inflammation contribute to salivary gland dysfunction in patients with chronic heart failure?. Front Immunol.

[23] Silness J., Löe H (1964). Periodontal disease in pregnancy II. Correlation between oral hygiene and periodontal condition. Acta Odontol. Scand.

[24] Mioshi E, Dawson K, Mitchell J (2006). The Addenbrooke's Cognitive Examination Revised (ACE-R): a brief cognitive test battery for dementia screening. Int J Geriatr Psychiatry.

[25] Prats-Sedano MA, Savulich G, Surendranathan A (2021). The revised Addenbrooke's Cognitive Examination can facilitate differentiation of dementia with Lewy bodies from Alzheimer's disease. Int J Geriatr Psychiatry.

[26] Yi Y, Ding L, Wen H (2020). Is Barthel Index Suitable for Assessing Activities of Daily Living in Patients With Dementia?. Front Psychiatry.

[27] Kawamura K, Murayama K, Takamura J (2022). Effect of a weekly functional independence measure scale on the recovery of patient with acute stroke: A retrospective study. Medicine (Baltimore).

[28] Medley A, Thompson M (2011). Development, reliability, and validity of the Sitting Balance Scale. Physiother Theory Pract.

[29] Nayak AR, Kashyap RS, Kabra D (2012). Time course of inflammatory cytokines in acute ischemic stroke patients and their relation to inter-alpha trypsin inhibitor heavy chain 4 and outcome. Ann Indian Acad Neurol.

[30] Dugue R, Nath M, Dugue A Roles of Pro- and Anti-inflammatory Cytokines in Traumatic Brain Injury and Acute Ischemic Stroke. 2017; InTech. doi: 10.5772/intechopen.70099.

[31] Zhang JM, An J (2007). Cytokines, inflammation, and pain. Int Anesthesiol Clin.

[32] Zhao H, Li Y, Zhang Y (2022). Role of Immune and Inflammatory Mechanisms in Stroke: A Review of Current Advances. Neuroimmunomodulation.

[33] Yu S, Cui W, Han J (2022). Longitudinal change of Th1, Th2, and Th17 cells and their relationship between cognitive impairment, stroke recurrence, and mortality among acute ischemic stroke patients. J Clin Lab Anal.

[34] Lappin MB, Campbell JD (2000). The Th1-Th2 classification of cellular immune responses: concepts, current thinking and applications in haematological malignancy. Blood Rev.

[35] Jin R, Liu L, Zhang S (2013). Role of inflammation and its mediators in acute ischemic stroke. J Cardiovasc Transl Res.

[36] Chen X, Oppenheim JJ (2014). Th17 cells and Tregs: unlikely allies. J Leukoc Biol.

[37] Sculley DV, Langley-Evans SC (2002). Salivary antioxidants and periodontal disease status. Proc Nutr Soc.

[38] Williamson S, Munro C, Pickler R (2012). Comparison of biomarkers in blood and saliva in healthy adults. Nurs Res Pract.

[39] Wang J, Liang Y, Wang Y (2013). Computational prediction of human salivary proteins from blood circulation and application to diagnostic biomarker identification. PLoS One.

[40] Zięba S, Maciejczyk M, Antonowicz B (2024). Comparison of smoking traditional, heat not burn and electronic cigarettes on salivary cytokine, chemokine and growth factor profile in healthy young adults-pilot study. Front Physiol.

[41] Bosch JA (2014). The use of saliva markers in psychobiology: mechanisms and methods. Monogr Oral Sci.

[42] Gerreth P, Gerreth K, Maciejczyk M (2021). Is an Oral Health Status a Predictor of Functional Improvement in Ischemic Stroke Patients Undergoing Comprehensive Rehabilitation Treatment?. Brain Sci.

[43] Sen S, Logue L, Logue M (2024). Dental Caries, Race and Incident Ischemic Stroke, Coronary Heart Disease, and Death. Stroke.

[44] Shahi S, Farhoudi M, Dizaj SM (2022). The Link between Stroke Risk and Orodental Status-A Comprehensive Review. J Clin Med.

[45] Shetty B, Fazal I, Khan SF (2023). Association between cardiovascular diseases and periodontal disease: more than what meets the eye. Drug Target Insights.

[46] Isola G, Polizzi A, Santonocito S (2022). Periodontitis activates the NLRP3 inflammasome in serum and saliva. J Periodontol.

[47] Polizzi A, Nibali L, Tartaglia GM (2024). Impact of nonsurgical periodontal treatment on arterial stiffness outcomes related to endothelial dysfunction: A systematic review and meta-analysis. J Periodontol.

[48] LaValley EA, Sen S, Mason E (2024). Dental Caries a Risk Factor for Intracerebral Hemorrhage. Cerebrovasc Dis.

[49] Chang Y, Woo HG, Lee JS (2021). Better oral hygiene is associated with lower risk of stroke. J Periodontol.

[50] Zheng X, Li X, Zhen J (2023). Periodontitis is associated with stroke. J Transl Med.

[51] Żukowski P, Maciejczyk M, Waszkiel D (2018). Sources of free radicals and oxidative stress in the oral cavity. Arch Oral Biol.

[52] Zięba S, Maciejczyk M, Zalewska A (2022). Ethanol- and Cigarette Smoke-Related Alternations in Oral Redox Homeostasis. Front Physiol.

[53] Tirandi A, Sgura C, Carbone F (2023). Inflammatory biomarkers of ischemic stroke. Intern Emerg Med.

[54] Siciliano M, Raimo S, Tufano D (2016). The Addenbrooke's Cognitive Examination Revised (ACE-R) and its sub-scores: normative values in an Italian population sample. Neurol Sci.

[55] Etemadi N, Holien JK, Chau D (2013). Lymphotoxin α induces apoptosis, necroptosis and inflammatory signals with the same potency as tumour necrosis factor. FEBS J.

[56] Clark IA, Alleva LM, Vissel B (2010). The roles of TNF in brain dysfunction and disease. Pharmacol Ther.

[57] Liberale L, Ministrini S, Carbone F (2021). Cytokines as therapeutic targets for cardio- and cerebrovascular diseases. Basic Res Cardiol.

[58] Yilmaz G, Arumugam TV, Stokes KY (2006). Role of T lymphocytes and interferon-gamma in ischemic stroke. Circulation.

[59] Zhu H, Hu S, Li Y (2022). Interleukins and Ischemic Stroke. Front Immunol.

[60] Peluzzo AM, Autieri MV (2022). Challenging the Paradigm: Anti-Inflammatory Interleukins and Angiogenesis. Cells.

[61] Tiwari M (2011). Science behind human saliva. J Nat Sci Biol Med.

[62] Kaufman E, Lamster IB (2002). The diagnostic applications of saliva-a review. Crit Rev Oral Biol Med.

[63] Xiao H, Wong DT (2011). Proteomics and its applications for biomarker discovery in human saliva. Bioinformation.

[64] Ghosh S, Dhobley A, Avula KK (2022). Role of Saliva as a Non-Invasive Diagnostic Method for Detection of COVID-19. Cureus.

[65] Lee YH, Wong DT (2009). Saliva: an emerging biofluid for early detection of diseases. Am J Dent.

[66] Biomarkers Definitions Working Group (2001). Biomarkers and surrogate endpoints: preferred definitions and conceptual framework. Clin Pharmacol Ther.

[67] Christensen H, Boysen G, Christensen E (2002). Plasma cytokines in acute stroke. J Stroke Cerebrovasc Dis.

[68] Beamer NB, Coull BM, Clark WM (1995). Interleukin-6 and interleukin-1 receptor antagonist in acute stroke. Ann Neurol.

[69] Kim HM, Shin HY, Jeong HJ (2000). Reduced IL-2 but elevated IL-4, IL-6, and IgE serum levels in patients with cerebral infarction during the acute stage. J Mol Neurosci.

[70] Tan S, Shan Y, Wang Y (2017). Exacerbation of oxygen-glucose deprivation-induced blood-brain barrier disruption: potential pathogenic role of interleukin-9 in ischemic stroke. Clinical Science.

[71] Gerreth P, Maciejczyk M, Zalewska A (2020). Comprehensive Evaluation of the Oral Health Status, Salivary Gland Function, and Oxidative Stress in the Saliva of Patients with Subacute Phase of Stroke: A Case-Control Study. J Clin Med.

[72] Maciejczyk M, Gerreth P, Zalewska A (2020). Salivary Gland Dysfunction in Stroke Patients Is Associated with Increased Protein Glycoxidation and Nitrosative Stress. Oxid Med Cell Longev.

[73] Smith AK, Kilaru V, Klengel T (2015). DNA extracted from saliva for methylation studies of psychiatric traits: evidence tissue specificity and relatedness to brain. Am J Med Genet B Neuropsychiatr Genet.

[74] Martin J, Kagerbauer SM, Gempt J (2018). Oxytocin levels in saliva correlate better than plasma levels with concentrations in the cerebrospinal fluid of patients in neurocritical care. J Neuroendocrinol.

[75] Thomas M, Knoblich N, Wallisch A (2018). Increased BDNF methylation in saliva, but not blood, of patients with borderline personality disorder. Clin Epigenetics.

[76] Browne RW, Kantarci A, LaMonte MJ (2013). Performance of multiplex cytokine assays in serum and saliva among community-dwelling postmenopausal women. PLoS One.

[77] Byrne ML, O'Brien-Simpson NM, Reynolds EC (2020). Acute phase protein and cytokine levels in serum and saliva: a comparison of detectable levels and correlations in a depressed and healthy adolescent sample. Brain Behav Immun. 2013;34:164-175. doi: 10.1016/j.bbi.2013.08.010. Erratum in: Brain Behav Immun.

[78] Parkin GM, Kim S, Mikhail A (2023). Associations between saliva and plasma cytokines in cognitively normal, older adults. Aging Clin Exp Res.

[79] Dawes C (1975). Circadian rhythms in the flow rate and composition of unstimulated and stimulated human submandibular saliva. J Physiol.

[80] Iorgulescu G (2009). Saliva between normal and pathological. Important factors in determining systemic and oral health. J Med Life.

[81] Kado I, Kunimatsu R, Yoshimi Y (2021). Surveillance of salivary properties of pre-orthodontic patients in relation to age and sex. Sci Rep.

[82] Kim J, Choi W, Kim KH (2024). Circadian Rhythms in Tongue Features. J Clin Med.

