# Supplementary material for: The screening assessment of pro-inflammatory, anti-inflammatory, Th1, Th2 and Th17 cytokines in saliva of patients with ischemic stroke
Source: Int J Med Sci. 2025 May 28;22(11):2686–99. doi: 10.7150/ijms.110452 (PMC12163425; doi:10.7150/ijms.110452)
Supplement: Supplementary file 1 — Supplementary table S1. [file ijmsv22p2686s1.pdf]

Table S1. General characteristics of both groups of stroke patients and controls.
ACE-R: Addenbrooke's Cognitive Examination Revised; BI: Barthel Index; DMFT: The Decayed, Missing and Filled Teeth; GI: Gingival Index; FIM: The Functional Independence Measure; PI: Plaque Index; SBS: Sitting Balance Scale.

**Characteristics**		**C (n =22)**			**Stroke (n =22)**			**P-value**
		Minimum	*Median*	*Maximum*	*Minimum*	*Median*	*Maximum*	
Age		53	65	80	53	65	80	> 0.9999
Sex	male n (%)	13 (59%)			13 (59%)			> 0.9999
	female n (%)	9 (41%)			9 (41%)			
Education	primary n (%)	2 (9%)			1 (4.5%)			> 0.9999
	vocational n (%)	8 (36.5%)			9 (41%)			
	secondary n (%)	8 (36.5%)			11 (50%)			
	university n (%)	4 (18%)			1 (4.5%)			
Place of residence	urban centre n (%)	5 (22.5%)			7 (32%)			> 0.9999
	small town n (%)	8 (36.5%)			10 (45.5%)			
	rural area or small village n (%)	9 (41%)			5 (22.5%)			
Houshold member(s)	with family member n (%)	20 (91%)			21 (95.5%)			> 0.9999
	none n (%)	2 (9%)			1 (4.5%)			
Comorbidities	hypertension n (%)	12 (54.5%)			11 (50%)			> 0.9999
	type 1 diabetes n (%)	0			0			
	type 2 diabetes n (%)	9 (41%)			8 (36.5%)			
	thyroid diseases n (%)	2 (9%)			2 (9%)			
	epilepsy n (%)	0			0			
	atrial fibrillation n (%)	4 (18%)			5 (22.5%)			
	limb thrombosis n (%)	4 (18%)			6 (27%)			
Drugs	< 5 drugs/day n (%)	19 (86.5%)			17 (77.5%)			> 0.9999
	> 5 drugs/day n (%)	3 (13.5%)			5 (22.5%)			
Dental examination								
PI		0	1.5	3	0	2	3	0.501
GI		0	2	2	0	1	2	0.1956
DMFT		9	32	32	9	30.5	32	0.7604

Cognitive and physical functional status					
ACE-R	ND	60	73	90	ND
BI	ND	1	9.5	14	ND
FIM	ND	0	62	105	ND
SBS	ND	0	10.5	20	ND
